# Long non‐coding RNA CRNDE promotes the proliferation, migration and invasion of hepatocellular carcinoma cells through miR‐217/*MAPK1* axis

**DOI:** 10.1111/jcmm.13856

**Published:** 2018-09-24

**Authors:** Haihao Wang, Ji Ke, Qiannan Guo, Kan‐Paatib Barnabo Nampoukime, Peiwen Yang, Ke Ma

**Affiliations:** ^1^ Division of Cardiothoracic and vascular surgery Tongji Hospital Huazhong University of Science and Technology Wuhan Hubei China; ^2^ Department of Forensic Science and Criminal Intelligence Hubei University of Police Wuhan Hubei China; ^3^ Reproductive Medicine Center Tongji Hospital Huazhong University of Science and Technology Wuhan Hubei China; ^4^ Division of Infectious Disease Tongji Hospital Huazhong University of Science and Technology Wuhan Hubei China

**Keywords:** CRNDE, hepatocellular carcinoma, LncRNA, MAPK1, MiR‐217

## Abstract

Hepatocellular carcinoma (HCC) is an invasive malignant tumour and the second major cause of cancer‐related deaths over the world. CRNDE and miR‐217 are non‐coding RNAs which play critical roles in cell growth, proliferation, migration. Mitogen‐activated protein kinase 1 (*MAPK1*) also participates in cancer cell process. Hence, this study aimed at investigating the effect of CRNDE on migration and invasion of HCC and figuring out the role of miR‐217 and *MAPK1* in this process. The overexpression of CRNDE was demonstrated by a microarray‐based lncRNA profiling study. CRNDE expression in HCC was verified by qRT‐PCR. MTT assay and BrdU staining were applied to detect cell proliferation level. Transwell assay was utilized to examine cell migration and invasiveness abilities. Wound healing assay was performed for further exploration of cell migration capacity. MiR‐217 was predicted by bioinformatics. The dual luciferase reporter assay was performed to corroborate the targeting relationship between CRNDE, miR‐217 and *MAPK1*. *MAPK1*, the downstream target of miR‐217, was predicted using bioinformatics and was further confirmed by qRT‐PCR and Western blot. The interaction between CRNDE, miR‐217 and *MAPK1* was studied by qRT‐PCR, Western blot, MTT, BrdU, transwell assay and wound healing assay. CRNDE was up‐regulated in HCC tissues and HCC cell lines. The high expression of CRNDE facilitated cell proliferation, migration and invasion, while the inhibited one affected on the contrary. MiR‐217, negatively correlated with CRNDE expression, was the target of CRNDE and was more lowly expressed in HCC. With the high expression of miR‐217, HCC cell proliferation, migration and invasion were suppressed. *MAPK1*, the possible target of miR‐217, was negatively correlated with miR‐217 but positively correlated with CRNDE and had the same effect in HCC formation process as CRNDE. Long non‐coding RNA CRNDE promotes the proliferation, migration and invasion of HCC cells through miR‐217/*MAPK1* axis.

## 1 INTRODUCTION

As the predominant liver malignancy, hepatocellular carcinoma (HCC) is the second major cause of cancer death in the world.^1^ In spite of remarkable efforts to discover novel therapeutic strategies against HCC, the long‐term survival rate remains dismal. Cancer metastasis and poor prognosis are two major obstacles in HCC therapy.^2^ Most of HCC patients still suffer from metastasis and recurrence after treatment. The molecular mechanism underlying HCC has not been clearly characterized. Thus, it is extremely critical to promote researches on molecular biomarkers which regulate metastatic behaviour and prognosis evaluation of HCC.^3^ Recently, an increasing number of studies have revealed the crucial role of non‐coding RNAs in HCC, including long non‐coding RNA (lncRNAs) and microRNAs (miRNAs).^4^, ^5^

LncRNAs are a class of poor conserved endogenous RNAs longer than 200 nucleotides that do not encode proteins but regulate gene expression.^6^ In recent years, accumulating evidences have revealed the importance of lncRNAs on cancer metastasis and development, suggesting the involvement of lncRNAs in cancer progression.^7^, ^8^ The lncRNA Colorectal Neoplasia Differentially Expressed (CRNDE), which was initially found to increase in colorectal cancer, was also overexpressed in various malignant diseases including HCC.^9^ Current evidences have suggested that CRNDE exert influence on the propagation and metastasis of malignant carcinoma.^10^ However, the expression profiles and precise biological functions of CRNDE in HCC remain largely unknown.

On the other hand, miRNAs, a class of endogenous non‐coding RNAs with 20‐25 nucleotides length, have been proved to modulate mRNA stability and protein translation.^11^ MiRNAs play a crucial role in cancer development process such as differentiation, proliferation and metastasis.^12^ According to previous studies, microRNA 217 (miR‐217) was linked with cell progression as a tumour suppressor.^13^, ^14^ But, the mechanism of miR‐217 is still not clear in HCC.

Epithelial‐to‐mesenchymal transition (EMT), the process by which epithelial cells gain migratory and invasive properties, has been identified as an important process in cancer progression, especially in HCC.^15^ Dysregulated expression of lncRNAs and miRNAs were reported to mediate the EMT progression in HCC cells.^16^, ^17^ Consequently, lncRNAs and miRNAs are considered as potential novel therapeutic targets against the poor prognosis and metastasis of HCC. Meanwhile, mitogen‐activated protein kinase 1 (*MAPK1*) is involved in a variety of cancer cell processes such as proliferation and migration.^18^ *MAPK1* has been proved to mediate EMT as an intracellular signalling molecule, and some signalling molecules can also affect EMT progression through MAPK1 pathway.^19^ Zhang et al found that miR‐217 regulated tumour growth and apoptosis by targeting the MAPK signalling pathway in colorectal cancer.^20^ Nevertheless, there are only a few reports about the interaction among CRNDE, miR‐217 and *MAPK1* in HCC cells.

Recently, some studies revealed that one potential function of lncRNAs was to directly interact with miRNAs, regulating their expression and activity.^21^ In recently described mechanism, lncRNAs might function as competitive endogenous RNAs to sponge specific miRNAs, thereby mediating the de‐repression of miRNAs targets.^22^ For instance, lncRNA MALAT1 facilitated migration and invasiveness by modulating miR‐1 in breast cancer.^23^ LncRNA H19 regulated cancer cell propagation by regulating miR‐194‐5p.^24^ LncRNA UCA1 exerted oncogenic effects by targeting mir‐193a‐3p in lung cancer.^25^ We therefore hypothesized that CRNDE might also directly interact with some particular miRNAs.

Herein, we reported that CRNDE and miR‐217 had different expression in HCC. Our results elucidated that CRNDE could modulate MAPK1 pathway by competitively inhibiting miR‐217, thereby promoting HCC cells migration and invasiveness. Our findings exhibited that CRNDE might serve as a potential therapeutic target against HCC.

## 2 MATERIALS AND METHODS

### 2.1 Patients and samples

HCC tissues were obtained from 46 patients with informed consents of Tongji Hospital. None of these patients received chemotherapeutic treatment or radical surgical treatment. All adjacent tissues and tumour tissues were preserved in liquid nitrogen under −80°C. This study was approved by the Institutional Ethics Committee of Tongji Hospital.

### 2.2 Microarray

Ten fresh human HCC tissues and paired para‐tumour tissues were acquired. Total RNA was extracted from these tissues and pooled. The collected RNA samples serve as templates for cDNA synthesis. Probe labelling and hybridization were carried out by Affymetrix GeneChip Human genome U133 plus 2.0 Array and the arrays were scanned by Affymetrix GeneChip Scanner 3000 7G (Affymetrix, California, USA). Then, we employed whole genome microarray expression profiling as a discovery platform to identify differentially expressed genes (DEGs) between HCC and normal control. After the preprocessing of the raw expression data, the DEGs were analysed using limma package in R/Bioconductor. The criteria for DEGs were based on fold change>2 combined with adjusted *P* value less than 0.05.

### 2.3 Cell lines and cultures

The HCC cell lines including HepG2, Huh‐7, HCCLM3, SNU449, SNU475, HepaRG and human normal hepatic cell line HL‐7702 were gained from BeNa Culture Collection (Beijing, China). HepG2, Huh‐7 and HCCLM3 cell lines were maintained in high‐glucose DMEM medium (Invitrogen, Carlsbad, CA, USA) with 10% foetal bovine serum (FBS, Invitrogen, CA, USA). HL‐7702, SNU449, SNU475 and HepaRG cells were cultured in RPMI‐1640 medium (GIBCO, Carlsbad, CA, USA) with 10% FBS (Invitrogen).

### 2.4 Cell transfection

PcDNA3.1‐CRNDE, sh‐CRNDE, pcDNA3.1‐MAPK1, sh‐MAPK1, miR‐217 mimics, anti‐miR‐217 and negative control were provided by GenePharma (Shanghai, China). Transfection of HepG2 and Huh‐7 cells was conducted using Lipofectamine^™^ 2000 (Invitrogen, Carlsbad, CA, USA). Transfected cells were cultured in 6‐well plates. After 48‐h cultivation, the cells were collected for subsequent analyses.

### 2.5 QRT‐PCR assay

Isolation of total RNA was conducted by TRIzol reagent (Invitrogen, Carlsbad, CA, USA). Quantitative reverse transcription PCR (qRT‐PCR) was performed using the THUNDERBIRD SYBR^®^ qPCR Mix (Toyobo, Japan). All reactions were run as follows: 94°C, 120 second; 94°C, 30 second; 56°C, 30 second; 72°C, 60 second; 30 cycles. Primer sequences were exhibited at Table 1.

**Table 1:** QRT‐PCR primer sequence

Name	Primer sequence (5′‐3′)
CRNDE	
Forward primer	ATATTCAGCCGTTGGTCTTTGA
Reverse primer	TCTGCGTGACAACTGAGGATTT
miR‐217	
Forward primer	TTGAGGTTGCTTCAGTGA
Reverse primer	GGAGTAGATGATGGTTAGC
MAPK1	
Forward primer	GGTGCCTCCTCTTGACTTCC
Reverse primer	AACCTGAACCTGACTGTCCATT
GADPH	
Forward primer	AACGGATTTGGTCGTATTG
Reverse primer	GGAAGATGGTGATGGGATT
U6	
Forward primer	CTCGCTTCGGCAGCACA
Reverse primer	AACGCTTCACGAATTTGCGT

### 2.6 Dual luciferase reporter assay

The wild‐type CRNDE and *MAPK1* 3′UTR sequence were amplified, and then, CRNDE‐mut, *MAPK1*‐mut, CRNDE‐wt and *MAPK1*‐wt were inserted into pmirGLO luciferase carrier (Promega, Madison, WI, USA). After that, pmirGLO‐CRNDE‐mut, pmirGLO‐CRNDE‐wt, mirGLO‐*MAPK1*‐mut, pmirGLO‐*MAPK1*‐wt or pmirGLO‐NC was transfected, respectively, into HepG2 or Huh‐7 cells with Lipofectamine^™^ 2000 (Invitrogen, Carlsbad, CA, USA). 100 nmol L^−1^ miR‐217 mimics and miR‐NC (GenePharma, Shanghai, China) were transfected subsequently into HepG2 or Huh‐7 cells. Luciferase activity was examined under the guidelines of the Dual Luciferase Reporter Assay System (Promega, Madison, WI, USA).

### 2.7 MTT assay

The cellular proliferation viability of HepG2 and Huh‐7 cells was determined by MTT (3‐(4, 5‐Dimethylthiazol2‐yl)‐2, 5‐Diphenyl Tetrazolium Bromide) assay. The HCC cells were planted in 96‐well plates and rendered quiescent by incubation in the serum‐free medium for 24 hour. Then, the old culture medium was removed and the fresh medium containing 5 mg/mL MTT (Sigma‐Aldrich, St. Louis, MO, USA) was added at 37°C. The MTT colorimetric assay was performed to detect cell proliferation after 1, 2, 3, 4, 5 day of incubation. The absorbance of resulting formazan crystals (solubilized with DMSO) was read at 490 nm on ELISA plate reader.

### 2.8 BrdU staining assay

Transfected cells were seeded on coverslips in 96‐well plates and cultured overnight. BrdU (10 μg/mL) was added to the culture medium and cells further incubated for 1 hour. Cells were immediately fixed in 4% paraformaldehyde for 10 minute and stained with an anti‐BrdU antibody (Biocompare, South San Francisco, CA) per manufacturer's instructions. The coverslips were counterstained with DAPI and imaged acquired with fluorescence microscopy (Olympus, Tokyo, Japan). Results were expressed as the cell number per field.

### 2.9 Transwell assay

HepG2 and Huh‐7 cells were suspended with fresh medium containing 1% FBS. For observing cell migration, cells were deposited in Transwell upper chambers (Corning Inc., Corning, NY, USA) and DMEM medium was added to lower chamber. For observing cell invasion, cells were seeded to the upper chamber coated with 50 matrigel gel (BD Bioscience, San Jose, CA, USA) and the bottom chamber was added with DMEM medium and 10% FBS. After fixed and stained for 30 minutes, cells were observed under a microscope.

### 2.10 Wound healing assay

Wound healing assay was performed for analysis of cell migration in vitro. Briefly, HepG2 and Huh‐7 cells were transfected with CRNDE, sh‐CRNDE, CRNDE+miR‐217 mimics, sh‐CRNDE+anti‐miR‐217, anti‐miR‐217, miR‐217 mimics, anti‐miR‐217+sh‐MAPK1, miR‐217 mimics+*MAPK1* and negative control. The HepG2 and Huh‐7 cells were cultured in 6‐well plates (5 × 10^5^/well) and incubated overnight. Culture inserts were removed after appropriate cell attachment and washed twice using PBS. Afterwards, cells were added in the DMEM medium with 10% FBS. At 0 and 24 hour after scratch would formation, images were obtained using an inverted microscope (Nikon, Tokyo, Japan) at a magnification of 40 × and were measured by Image Pro Plus software (Media Cybernetics, Inc., Rockville, MD, USA).

### 2.11 Western blot

After washed with PBS, cells were lysed with RIPA lysate (Beyotime, Shanghai, China). Protein concentrations were determined using Pierce BCA Protein Assay Kit (Pierce, Rockford, IL, USA). Protein was resolved by sodium dodecyl sulphate‐polyacrylamide gel electrophoresis (SDS‐PAGE) and electrophoretically transferred to polyvinylidene fluoride (PVDF) membrane (Invitrogen, USA). Afterwards, membranes were incubated with primary antibodies (Anti‐E‐Cadherin, ab1416, 1:50; Anti‐Mucin‐1, ab109185, 1:1000; Anti‐Vimentin, ab8978, 1/100; Anti‐Fibronectin, ab23750, 1 μg/mL; Anti‐c‐Myc, ab39688, 1/500; Anti‐β‐Actin, ab11003, 1/500, Abcam, Cambridge, MA, USA; Anti‐MAPK1, 1:100, Bosterbio, USA). Subsequently, we added IgG‐HRP labelled goat anti‐mouse secondary antibody (ab205719, 1:10000, Abcam, Cambridge, MA, USA). The immunoreactive proteins were visualized using the ECL Detection System (Life technologies, Gaithersburg, MD, USA).

### 2.12 Statistical analysis

Mean ± standard deviation (SD) presented all quantitative values. Student's *t* test was utilized for comparison between two groups. Paired *t* test was used for CRNDE/miR‐147 comparison between adjacent and tumour tissues, unpaired *t* test was applied for other comparison between two groups. One‐way analysis of variance (ANOVA) was applied for comparison in multi‐groups. Statistical analyses were conducted by GraphPad Prism v6.0 (Graphpad Software, La Jolla, CA, USA). The difference was statistically significant when *P* < 0.05.

## 3 RESULTS

### 3.1 CRNDE was screened out by microarray analysis

**Figure 1:**
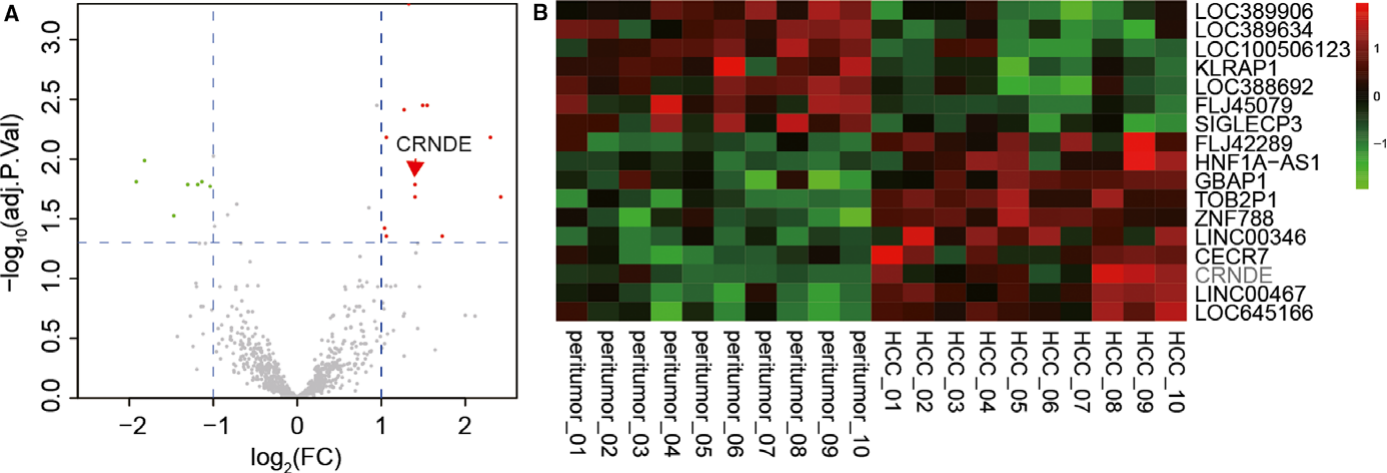
CRNDE was screened out by bioinformatics analysis. (A) The volcanic plot showed that CRNDE was significantly overexpressed in hepatocellular carcinoma (*P* < 0.001). (B) The heat map showed the significantly highly or lowly expression genes in HCC tissues, and CRNDE was sharply up‐regulated in HCC tissues

Ten pairs of HCC tissues and adjacent tissues were used to perform microarray. Fold change > 2 and *P* < 0.05 were applied to explore the abnormal lncRNA expressions. The volcanic plot and the heat map of lncRNA expression reflected that compared with adjacent normal tissues, CRNDE was up‐regulated in HCC tumour tissues. (Figure 1A and B).

### 3.2 CRNDE highly expressed while miR‐217 had a low expression in HCC cells and there was a targeted regulatory relationship between them

**Figure 2:**
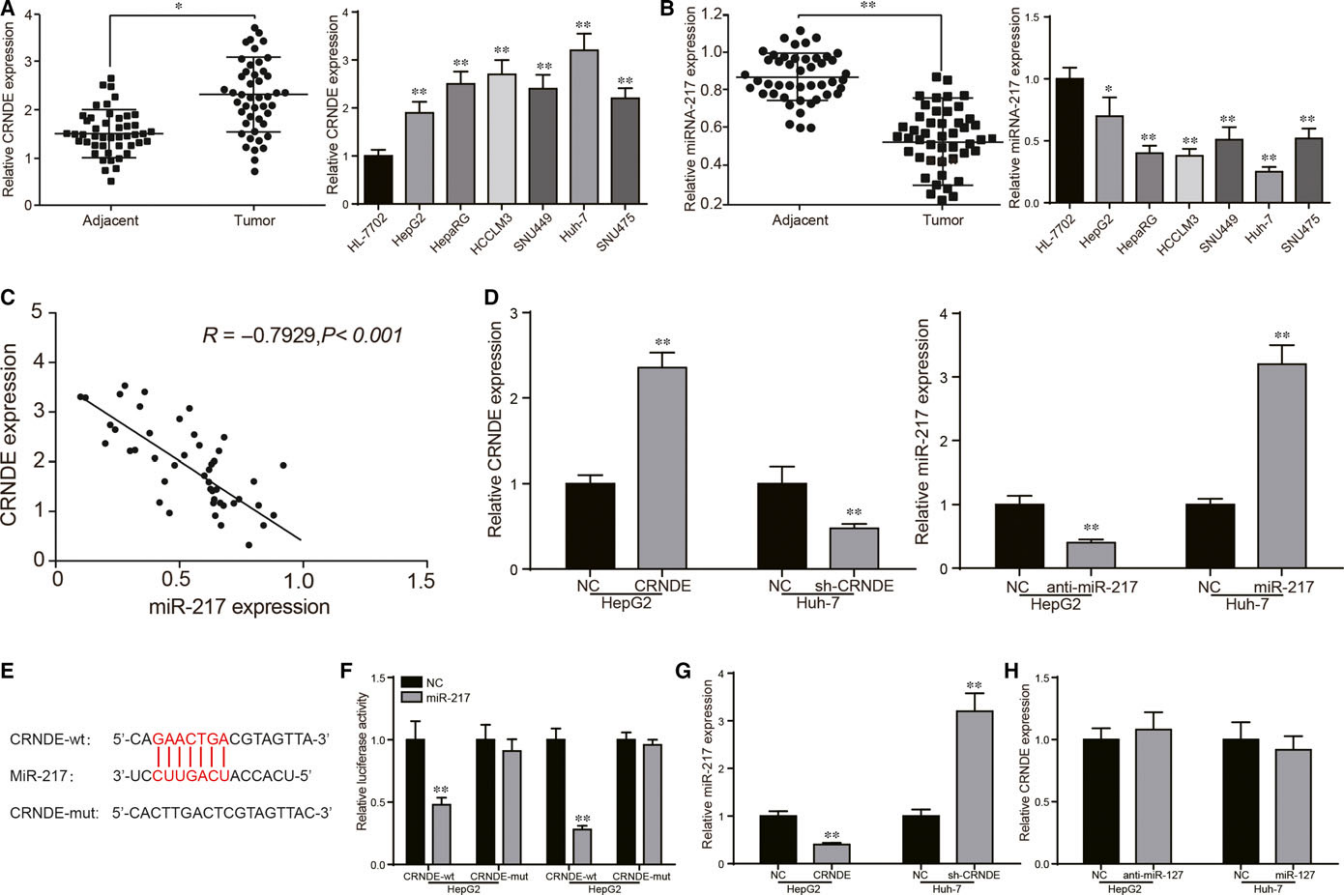
Circumstance of CRNDE/miR‐217 expression in vitro/*vivo* and the relationship between them. (A) CRNDE expression in tissues and HCC cells. (B) MiR‐217 expression in tissues and HCC cells. (C) Pearson's correlation analysis revealed CRNDE expression was negatively related with miR‐217 expression in HCC tissues. (D) Transfection efficiencies test of CRNDE/sh‐CRNDE and miR‐217/anti‐miR‐217 verified by qRT‐PCR. (E) The sequence of binding sites between CRNDE and miR‐217, and the sequence of mutant CRNDE. (F) Dual luciferase reporter assays showed that miR‐217 could regulate the luciferase activity of CRNDE‐WT, rather than CRNDE‐Mut. (G) QRT‐PCR showed that CRNDE could negatively regulate miR‐217 expression in HCC cells. CRNDE could significantly decrease the expression of miR‐217 in HepG2 cells while sh‐CRNDE could significantly increase the expression of miR‐217 in Huh‐7 cells. (H) MiR‐217 could not inversely regulate CRNDE expression in HCC cells. *P < 0.05, **P < 0.01, compared with adjacent/HL‐7702/NC group

The overexpression of CRNDE was further confirmed by qRT‐PCR in 46 HCC specimens (Figure 2A, *P* < 0.05). The CRNDE was also highly expressed in various liver cancer cell lines compared with the one in normal hepatocyte HL‐7702. Among HCC cell lines, CRNDE was relatively more highly expressed in Huh‐7 cells and more lowly expressed in HepG2 cells (Figure 2A, *P* < 0.01). Huh‐7 and HepG2 cell lines were used in follow‐up assays. The correlation between CRNDE and clinicopathologic characteristic of patients is shown in Table 2. Chi‐square test demonstrated that CRNDE had a close relation with AJCC stage and cancer progression like migration and invasion. Among these 46 HCC samples, miR‐217 was detected down‐regulated in tumour tissues (Figure 2B, *P* < 0.01). The miR‐217 was also lowly expressed in various liver cancer cell lines compared with the one in normal hepatocyte HL‐7702 (Figure 2B, *P* < 0.05, *P* < 0.01). Pearson's correlative analysis suggested miR‐217 expression was negatively correlated with CRNDE expression (Figure 2C, *P* < 0.001). The transfection efficiency test was applied for further experiments. QRT‐PCR results showed that CRNDE was significantly changed after the transfection of CRNDE or sh‐CRNDE while miR‐217 expression level was conspicuously fluctuated after the transfection of anti‐miR‐217 or miR‐217 mimics (Figure 2D, *P* < 0.01). Bioinformatics and dual luciferase reporter assay demonstrated that miR‐217 was the target of CRNDE (Figure 2E and F, *P* < 0.01). The up‐regulated CRNDE decreased miR‐217 expression in HepG2 cells, while the inhibited CRNDE dramatically increased miR‐217 expression in Huh‐7 cells (Figure 2G, *P* < 0.01). However, different miR‐217 expression levels scarcely influenced CRNDE expression level (Figure 2H). Based on above results, we hypothesized that the development of hepatocellular carcinoma might be closely related to the overexpression of CRNDE and low expression of miR‐217. Based on targeted regulatory relationship between CRNDE and miR‐217 was confirmed, we speculated CRNDE might affect HCC process via regulating miR‐217.

**Table 2:** Correlation between CRNDE expression and clinicopathologic characteristics in 46 cases of HCC tissues

Characteristics total cases	N of cases 46	CRNDE expression		*P* value^a^
		Low	High	
Age (years)				
≤60	30	15	15	0.7628
>60	16	9	7	
Gender				
Male	27	16	11	0.3695
Female	19	8	11	
AJCC stage				
I	14	10	4	0.0104*
II	21	13	8	
III	7	1	6	
IV	4	0	4	
Tumour size				
≤3 cm	37	22	15	0.0660
>3 cm	9	2	7	
Vascular invasion				
Yes	15	3	12	0.0122*
No	31	19	12	
Distant metastasis				
M_0_	27	18	9	0.0031**
M_1_	19	4	15	

*P < 0.05, **P < 0.01; AJCC, American Joint Committee on Cancer.

a Chi‐square test.

### 3.3 CRNDE could enhance proliferation, migration and invasion through the promotion of EMT process via regulating miR‐217 in HCC cells

**Figure 3:**
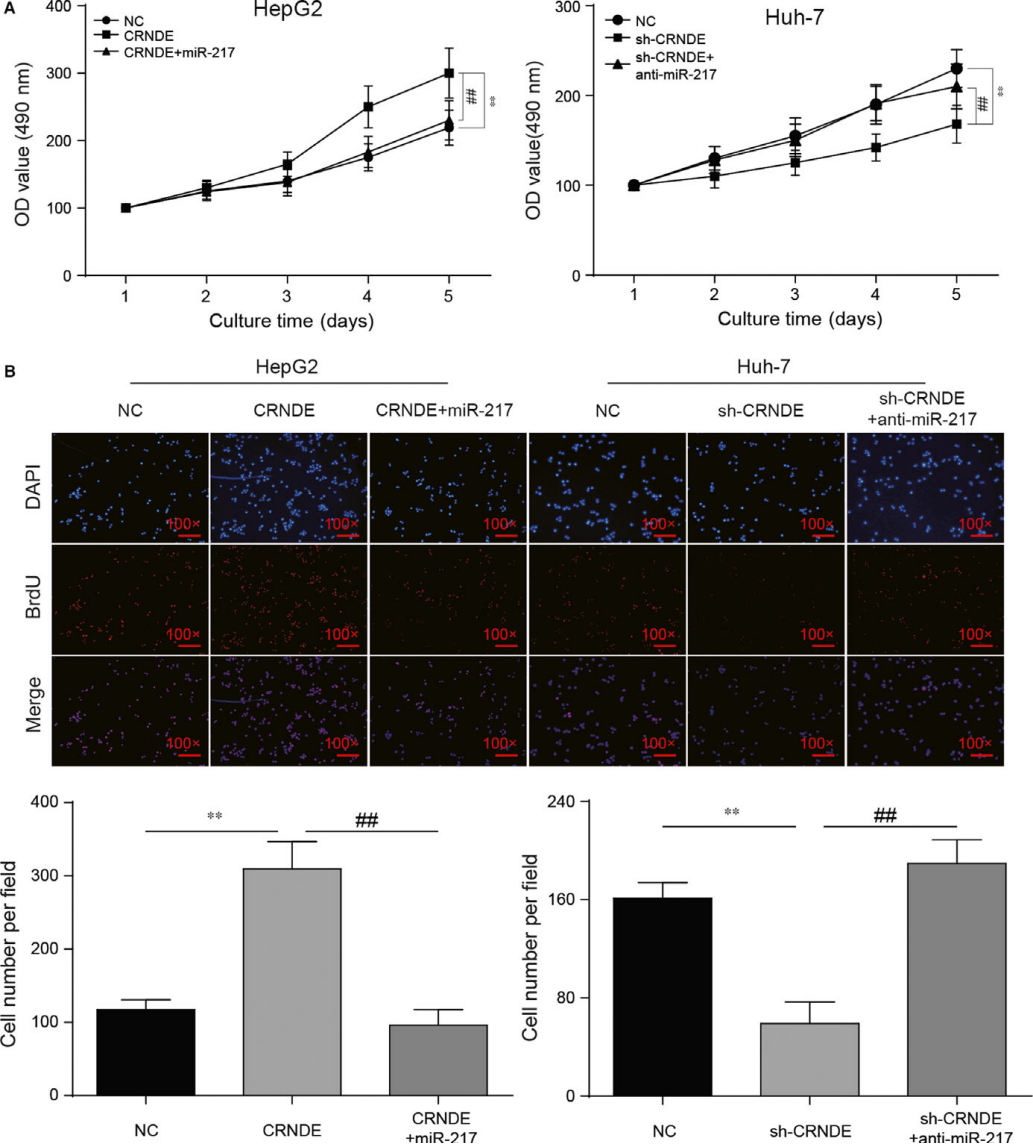
CRNDE/miR‐217 played a role in regulating HCC cell proliferation. (A) MTT assay showed CRNDE and down‐regulated miR‐217 could accelerate cell proliferation while down‐regulated CRNDE and miR‐217 could slow down cell proliferation. (B) BrdU staining assay showed the cell propagation condition in different transfection groups. **P < 0.01, compared with NC group. ^##^ *P* < 0.01, compared with CRNDE+miR‐217/sh‐CRNDE+ anti‐miR‐217 group

MTT assay was applied to detect the proliferation level while BrdU staining assay was utilized for further verification of cell propagation ability. MTT assay showed that cell proliferation level was increased by CRNDE and the condition was offset with the addition of miR‐217 mimics. In sh‐CRNDE group, cell proliferation was significantly restrained and the viability of proliferation was recovered with the addition of anti‐miR‐217 (Figure 3A, *P* < 0.01). BrdU staining assay showed that cell number per unit area was increased after the transfection of CRNDE and the cell quantity was restored by miR‐217 mimics. In Huh‐7 cells, sh‐CRNDE could significantly decrease the cell number and this condition could be eliminated by anti‐miR‐217 (Figure 3B, *P* < 0.01).

**Figure 4:**
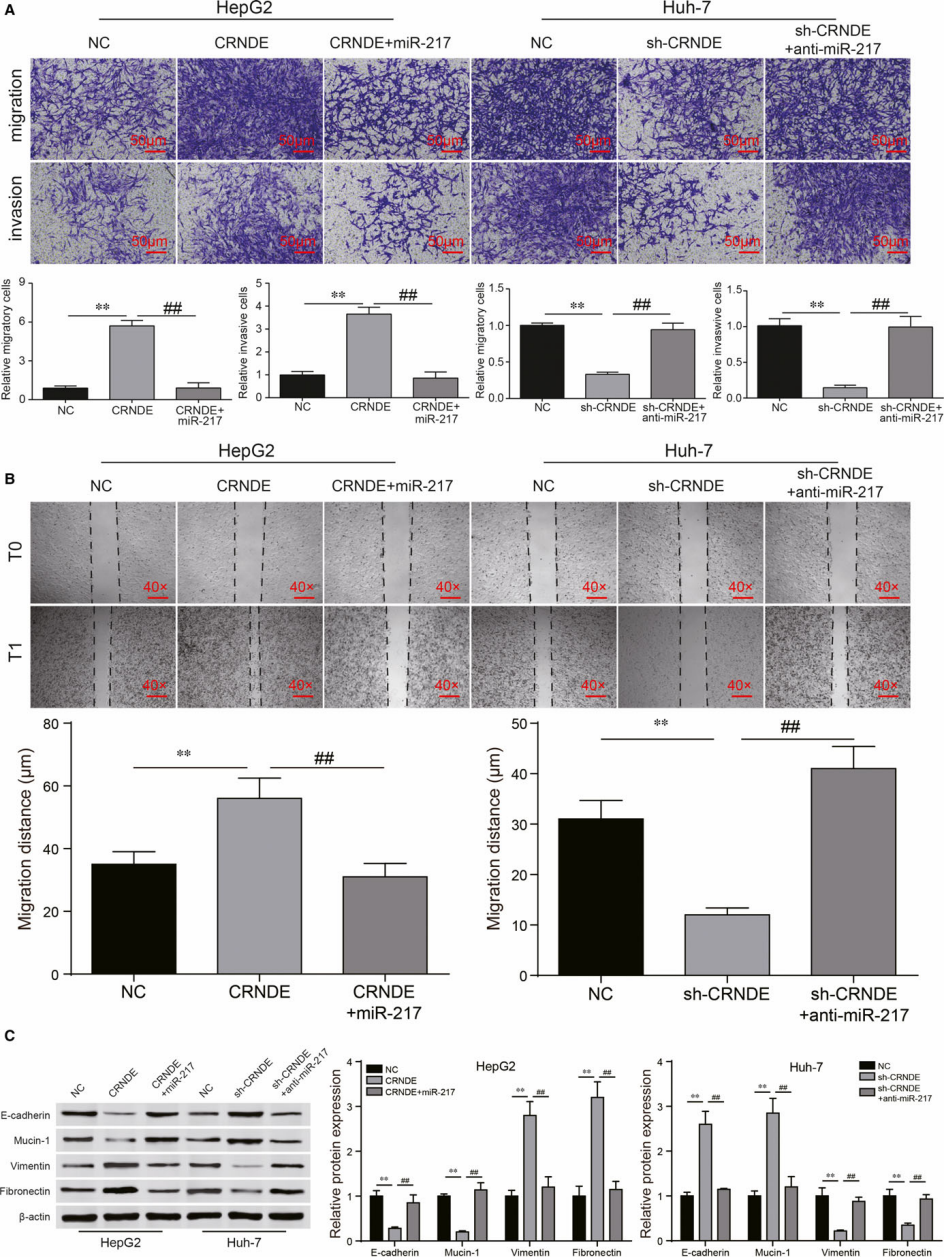
CRNDE/miR‐217 took part in cell migration, invasion and EMT regulatory process. (A) Transwell assay showed that CRNDE and down‐regulated miR‐217 could enhance cell migration and invasion abilities, down‐regulated CRNDE and miR‐217 could weaken capacities of cell migration and invasion. (B) Wound healing assay was performed for further verification of cell migration abilities. (C) Western blot exhibited the protein expression levels of epithelial makers E‐cadherin/Mucin‐1 were reduced by CRNDE and anti‐miR‐217. Besides, the results were conversed by miR‐217 and sh‐CRNDE. Furthermore, the protein expression levels of mesenchymal markers Vimentin/Fibronectin were increased following the EMT activation. **P < 0.01, compared with NC group. ^##^ *P* < 0.01, compared with CRNDE+miR‐217/sh‐CRNDE+ anti‐miR‐217 group

Transwell assay and wound healing assay were applied to detect the migration and invasion levels while Western blot was utilized to verify the EMT process of HCC cells. The up‐regulation of CRNDE significantly promoted migration and invasive abilities of HepG2 cells. With the addition of miR‐217 mimics, the metastatic capabilities were significantly weakened (Figure 4A, *P* < 0.01). In Huh‐7 cells, sh‐CRNDE conspicuously decreased cell migration and invasion levels. After the transfection of anti‐miR‐217, this condition was revered (Figure 4A, *P* < 0.01). Wound healing assay further proved CRNDE could accelerate cell migration and miR‐217 would block cell metastasis (Figure 4B, *P* < 0.01). Generally, loss of E‐cadherin/Mucin‐1 and activation of Vimentin/Fibronectin expression are considered to be the fundamental events in EMT. As shown in Figure 4C, overexpressed CRNDE observably decreased E‐cadherin/Mucin‐1 protein levels and increased Vimentin/Fibronectin protein levels (*P* < 0.01), whereas the miR‐217 affected on the contrary (*P* < 0.01). Sh‐CRNDE conspicuously increased E‐cadherin/Mucin‐1 protein levels and decreased Vimentin/Fibronectin protein levels (*P* < 0.01), whereas the anti‐miR‐217 had an opposite effect (*P* < 0.01). Therefore, CRNDE enhance proliferation, migration and invasion by promoting EMT process via regulating miR‐217 in HCC cells was evidenced preliminarily.

### 3.4 *MAPK1* was the target of miR‐217 and was regulated by CRNDE/miR‐217 axis

**Figure 5:**
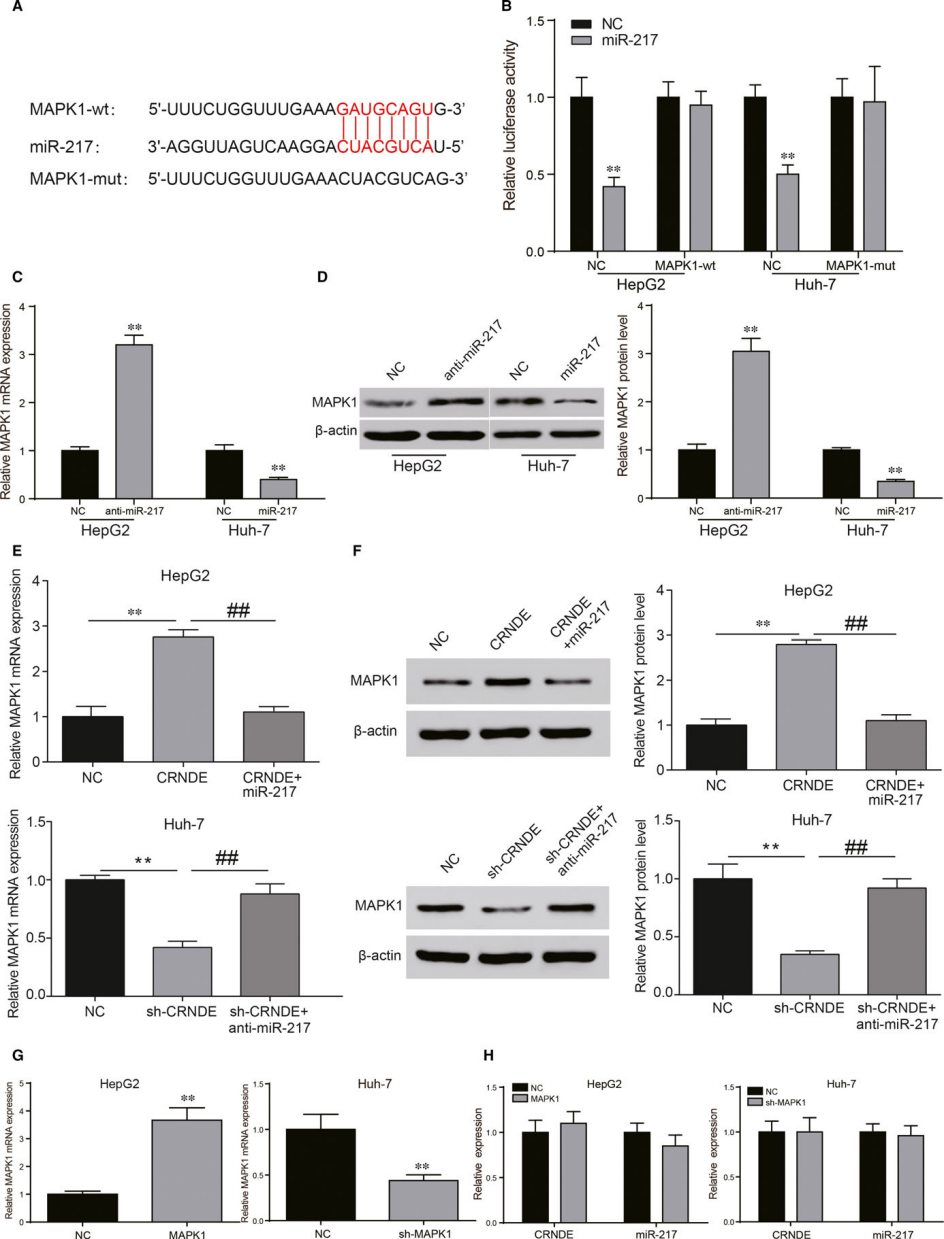
*MAPK1* was a target of miR‐217 and positively regulated by CRNDE in HCC cells. (A) The sequence of binding sites between *MAPK1* and miR‐217, and the sequence of mutant *MAPK1*. (B) Dual luciferase reporter assays showed miR‐217 regulated the luciferase activity of *MAPK1*‐wt, rather than *MAPK1*‐mut in HCC cells. (C, D) Quantitative real‐time PCR and Western blot analysis revealed that miR‐217 inhibitors could significantly increase the expression of *MAPK1* in HepG2 cells. MiR‐217 mimics could significantly decrease the expression of *MAPK1* in Huh‐7 cells. (E, F) MiR‐217 restoration reduced the high expression of *MAPK1* in CRNDE‐overexpressing HepG2 cells. MiR‐217 knockdown increased the low expression of *MAPK1* in CRNDE‐knockout Huh‐7 cells. (G) Transfection efficiencies test verified by qRT‐PCR. (H) *MAPK1* could not inversely regulate up‐stream CRNDE/miR‐217 expression in HCC cells. **P < 0.01, compared with NC group. ^##^ *P* < 0.01, compared with CRNDE+miR‐217/sh‐CRNDE+anti‐miR‐217 group

Bioinformatics analysis was utilized to predict that *MAPK1* was the possible targeting genes of miR‐217 (Figure 5A). Dual luciferase reporter assay demonstrated that miR‐217 mimics could regulate the luciferase activity of *MAPK1*‐wt, but had no obvious effect on *MAPK1*‐mut (Figure 5B, *P* < 0.01). HepG2 transfected with miR‐217 inhibitors and Huh‐7 transfected with miR‐217 mimics were used to test the influence of miR‐217 on *MAPK1*. The qRT‐PCR and Western blot results suggested that miR‐217 negatively regulated *MAPK1* expression, *MAPK1* was regulated by miR‐217 (Figure 5C and D, both *P* < 0.01). In order to further investigate the role of miR‐217 and *MAPK1*, HepG2 cells were transfected with extra CRNDE, raising *MAPK1* expression. More importantly, CRNDE+miR‐217 mimics group reversed the up‐regulation of *MAPK1* (Figure 5E and F, both *P* < 0.01). Conversely, sh‐CRNDE+anti‐miR‐217 group reversed the down‐regulation of *MAPK1* reduced by sh‐CRNDE group in Huh‐7 cells (Figure 5E and F, both *P* < 0.01). Transfection efficiency test showed *MPAK1* expression level was significantly changed after the transfection of *MAPK1* or sh‐MAPK1 (Figure 5G, *P* < 0.01). To investigate whether *MAPK1* can reversely regulate up‐stream CRNDE/miR‐217 expression, the qRT‐PCR was applied. The qRT‐PCR results of CRNDE/miR‐217 demonstrated that different *MAPK1* expression levels scarcely influenced up‐stream CRNDE/miR‐217 expression level (Figure 5H).

### 3.5 MiR‐217/*MAPK1* regulatory axis affected HCC cell proliferation, migration, invasion enhance proliferation, migration and invasion through EMT process

**Figure 6:**
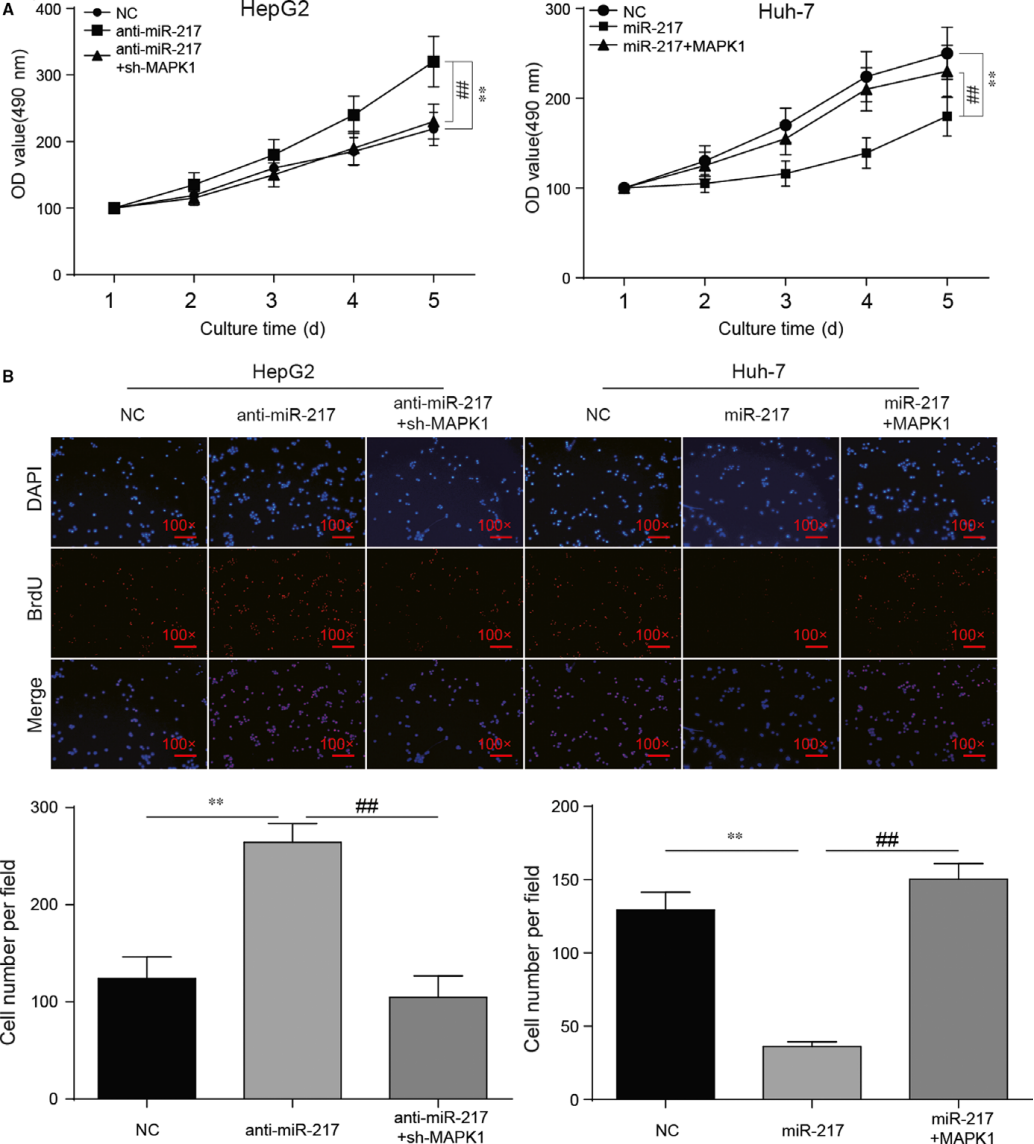
*MAPK1* played a role in HCC cell proliferation through miR‐217 regulation. (A) MTT assay showed that sh‐MAPK1 could largely reverse the accelerating effects of anti‐miR‐217 on proliferation of HepG2 cells while *MAPK1* abrogated the suppressive effects of miR‐217 mimics on proliferation of Huh‐7 cells. (B) BrdU staining assay demonstrated HCC cell proliferation ability was enhanced by anti‐miR‐217 or *MAPK1* while it was weakened by sh‐MAPK1 or miR‐217. **P < 0.01, compared with NC group. ^##^ *P* < 0.01, compared with anti‐miR‐217+ sh‐MAPK1/miR‐217+*MAPK1* group

MTT assay showed that HepG2 cell proliferation level was increased in anti‐miR‐217 group and the condition was offset with the addition of sh‐MAPK1. In Huh‐7 cells, cell proliferation was significantly restrained by miR‐217 mimics and the viability of proliferation was recovered with the addition of *MAPK1* (Figure 6A, *P* < 0.01). BrdU staining assay showed that cell number per unit area was increased after the transfection of anti‐miR‐217 and the cell quantity was restored by sh‐MAPK1. In Huh‐7 cells, miR‐217 could significantly decrease the cell number and this condition could be eliminated by *MAPK1* (Figure 6B, *P* < 0.01).

**Figure 7:**
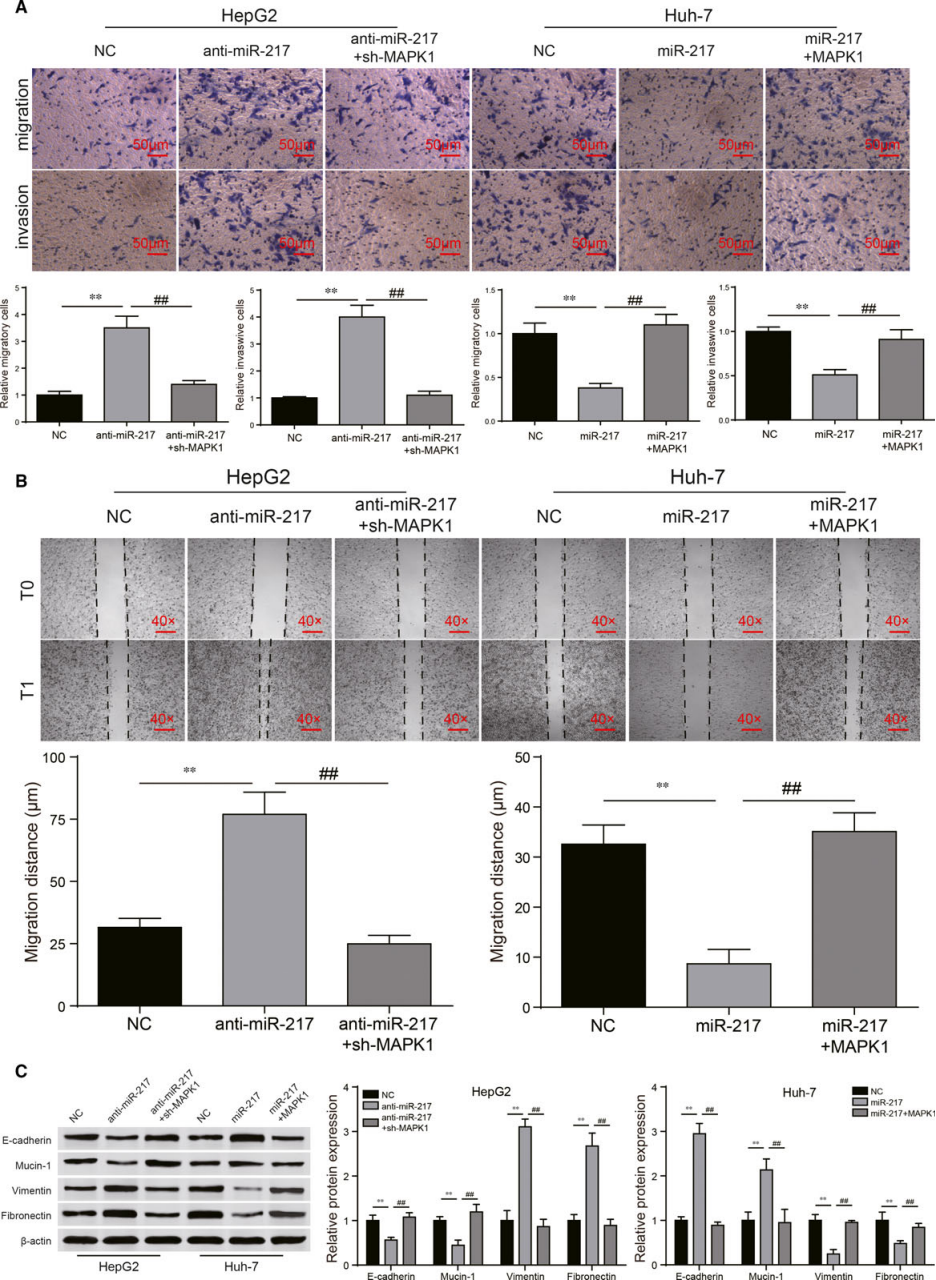
MiR‐217/*MAPK1* regulated cell migration, invasion and EMT regulatory process in HCC cells. (A) Transwell assays showed that sh‐MAPK1 could largely reverse the promoting effects of anti‐miR‐217 on migration and invasion of HepG2 cells. *MAPK1* could abrogate the suppressive effects of miR‐217 mimics on migration and invasion of Huh‐7 cells. (B) Wound healing assay was performed for further verification of cell migration abilities. (C) Sh‐MAPK1 could largely reverse the promoting effects of anti‐miR‐217 on EMT progression of HepG2 cells while *MAPK1* could abrogate the suppressive effects of miR‐217 mimics on EMT progression of Huh‐7 cells. **P < 0.01, compared with NC group. ^##^ *P* < 0.01, compared with anti‐miR‐217+sh‐MAPK1/miR‐217+*MAPK1* group

Transwell assay and wound healing assay showed that down‐regulation of miR‐217 significantly promoted migration and invasive abilities of HepG2 cells. With the addition of sh‐MAPK1, the metastatic capabilities were significantly weakened (Figure 7A and B, *P* < 0.01). In Huh‐7 cells, miR‐217 conspicuously decreased cell migration and invasion levels. After the transfection of *MAPK1*, this condition was revered (Figure 7A and B, *P* < 0.01). Besides, down‐regulated miR‐217 observably decreased E‐cadherin/Mucin‐1 protein levels and increased Vimentin/Fibronectin protein levels, whereas sh‐MAPK1 affected on the contrary (Figure 7C, *P* < 0.01). MiR‐217 conspicuously increased E‐cadherin/Mucin‐1 protein levels and decreased Vimentin/Fibronectin protein levels, whereas the *MAPK1* generated an opposite effect (Figure 7C, *P* < 0.01). To sum up, miR‐217/*MAPK1* regulatory axis played a role in regulating HCC progress.

### 3.6 CRNDE/miR‐217 were related with the expression of EMT markers and participated in mediating MAPK1 signalling

**Figure 8:**
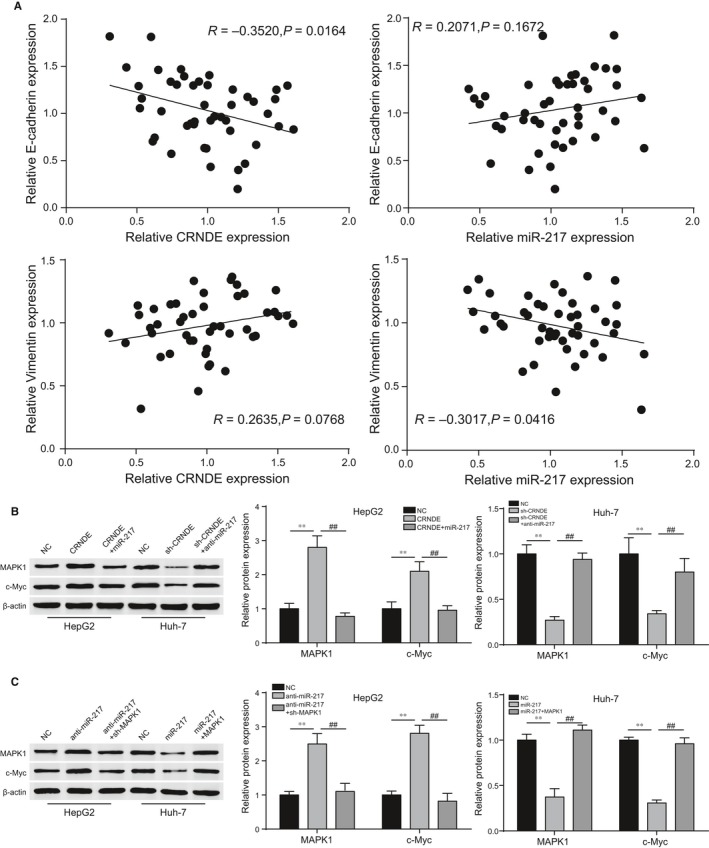
CRNDE/miR‐217 were related to EMT process and MAPK1 signalling pathway. (A) Expression of EMT markers was related with CRNDE/miR‐217 expression. (B, C) The protein expression levels of *MAPK1* and down‐stream *c‐Myc* were regulated by CRNDE/miR‐217 axis. **P < 0.01, compared with NC group. ^##^ *P* < 0.01, compared with anti‐miR‐217+sh‐MAPK1/miR‐217+*MAPK1* group

Pearson correlative analysis suggested the expression of EMT marker E‐cadherin was negatively correlated with CRNDE expression but positively correlated with miR‐217 expression. Vimentin, the mesenchymal marker of EMT, was positively correlated with CRNDE expression while negatively related to miR‐217 (Figure 8A). The results demonstrated that CRNDE/miR‐217 had a close relation with EMT process. As for MAPK1 signalling, the protein levels of *MAPK1* and its down‐stream *c‐Myc* were detected by Western blot with the different treatments. CRNDE or down‐regulated miR‐217 could significantly enhance *MAPK1*/*c‐Myc* protein expression and the potentiation could be eliminated by miR‐217 or sh‐MAPK1 (Figure 8B, *P* < 0.01). Down‐regulated CRNDE and miR‐217 conspicuously reduced *MAPK1*/*c‐Myc* protein expression, and the inhibiting effect was removed by anti‐miR‐217 or *MAPK1* (Figure 8C, *P* < 0.01). Combined the results, it was proved that MAPK1 signalling was mediated by CRNDE/miR‐217 regulatory axis.

## 4 DISCUSSION

HCC has been regarded as a common malignant tumour for high recurrence and poor prognosis.^2^ Targeting the metastasis of HCC cells has become a therapeutic object for developing novel HCC treatments. So far, mounting evidences have indicated that lncRNAs had close connection with the HCC progression.^26^ For instance, Lan et al have reported that HCC tumorigenesis was accelerated by lncRNA PVT1.^27^ In addition, Lu et al proved that overexpressed lncRNA HOXD‐AS1 was a critical regulator of metastasis in HCC.^28^ CRNDE has been identified to promote tumour development,^9^, ^29^ and its function in HCC remains had been preliminarily revealed in some research, such as Chen et al revealed that CRNDE promotes hepatic carcinoma cell proliferation, migration and invasion by suppressing miR‐384.^30^ In this study, CRNDE expression was proved to be remarkably increased in HCC and the results are consistent with previous studies. Up till now, a number of lncRNAs have been characterized as regulators in HCC cells metastasis process.^31^ In in vitro experiments, we found that CRNDE facilitated HCC cells metastasis and promoted the EMT progression by decreasing E‐cadherin/Mucin‐1 and increasing Vimentin/Fibronectin. Thus, we concluded that CRNDE functioned as a tumour promoter by accelerating metastasis and EMT progression of HCC cells.

Increasing studies have reported that abnormally expressed lncRNAs can function as competitive endogenous RNA (ceRNA) to modulate miRNAs and play key roles in the occurrence and development of malignant tumour.^32^, ^33^ In our study, bioinformatics analysis and luciferase reporter assay verified that miR‐217 was a target of CRNDE in HCC cells. By modulating expression levels of CRNDE and miR‐217, we found that CRNDE negatively mediated miR‐217 expression, while the dysregulated miR‐217 did not influence CRNDE expression reversely. According to the ceRNA theory, we supposed that CRNDE could act as a sponge to negatively regulate miR‐217, exerting its promoting effects on HCC cells via modulating miR‐217. Previous study Yu et al discovered that CRNDE involves in the cell proliferation, migration and invasion of colorectal cancer cells via increasing the expression of *TCF7L2* and activity of Wnt/β‐catenin signalling through binding miR‐217 competitively^34^ and the consequence was consisted with our experimental results.

To further investigate the molecular network of miR‐217, we investigated *MAPK1*, a downstream target of miR‐217. Previous studies have confirmed that increased *MAPK1* expression could function as tumour promoter in HCC.^35^, ^36^ Tang et al demonstrated that *MAPK1* was capable to regulate cell propagation and metastasis in ovarian cancer.^37^ Li et al reported the effect of *MAPK1* gene on metastasis and EMT progression in cervical cancer.^19^ Consistently, our results showed that *MAPK1* down‐regulated by miR‐217 facilitated metastasis and EMT process of HCC cells, indicating that miR‐217 suppressed HCC via negatively modulating *MAPK1* expression.

Generally speaking, lncRNAs can act as miRNAs sponges to reduce their regulatory effect on specific mRNAs. Zhu et al reported that lncRNA H19 sponged miR‐675 and repressed prostate cancer metastasis by targeting TGFB1.^38^ Huan et al revealed that lncRNA CRNDE activated Wnt/β‐catenin pathway through acting as sponge of miR‐136 in breast cancer.^39^ We did experiments to further explore the underlying mechanisms involved in CRNDE, miR‐217 and *MAPK1*, and we found that CRNDE could positively regulate the expression of *MAPK1*, but this increase was reversed by miR‐217 restoration. Moreover, the knockdown or restoration of miR‐217 also altered the positive effects of CRNDE on metastasis and EMT progression in HCC cells. Therefore, based on our results and existed literatures, we concluded that CRNDE participated in the regulation of *MAPK1* through interacting with miR‐217, which exert influence on HCC process.

As for MAPK1 signalling pathway, we detected *MAPK1* and down‐stream *c‐Myc* protein expression levels by Western blot. The results showed that CRNDE could promote MAPK1 signalling while miR‐217 worked on the contrary. There was a lot of evidences had proved that MAPK1 signalling and its down‐stream pathway played a role in regulating proliferation, migration, invasion and EMT. Hermeking et al identified that the cyclin‐dependent kinase 4 (*CDK4*) gene as a transcriptional target of *c‐Myc*. The prototypic oncogene *c‐Myc* encodes a transcription factor that can drive proliferation by promoting downstream cell cycle pathway.^40^ Furthermore, Shi et al proved c‐Myc was the major transcription factor promoting *VEGF* expression,^41^ Fantozzi et al discovered VEGF signalling‐mediated angiogenesis links EMT‐induced cancer stemness to tumour initiation.^42^ *VEGF* promoted tension‐independent FAK signalling activation which played an important role in cell migration and invasion.^43^ Above all, MAPK1 signalling was connected to downstream cell cycle, VEGF and FAK signalling which was known by regulating proliferation, metastasis and EMT process. Combined with the result of our study, it was proved that CRNDE/miR‐217 mediated HCC progress via MAPK1 signalling pathway. However, to well elucidate the pathogenesis of HCC, some deficiencies in our study should be ameliorated in future researches. For example, in vivo experiment should be employed to verify the molecular mechanism of CRNDE/miR‐217/*MAPK1* axis.

In conclusion, this study elucidated that CRNDE was increased in HCC and promoted migration, invasiveness and EMT progression of HCC cells via CRNDE/miR‐217/*MAPK1* axis. Specifically, CRNDE could promote HCC process as a molecular sponge of miR‐217, which targeting MAPK1 pathway. We preliminarily clarified the relationship between CRNDE and miR‐217. Promising therapeutic strategies against HCC might be established by targeting CENDE or repairing the balance between miR‐217 and *MAPK1*.

## CONFLICTS OF INTEREST

The authors confirm that there are no conflicts of interest.

## References

[1] Torre LA , Bray F , Siegel RL , et al. Global cancer statistics, 2012. CA Cancer J Clin. 2015;65:87‐108. 25651787 10.3322/caac.21262

[2] Donadon M , Solbiati L , Dawson L , et al. Hepatocellular carcinoma: the role of interventional oncology. Liver Cancer. 2016;6:34‐43. 27995086 10.1159/000449346 PMC5159721

[3] Peng C , Hu W , Weng X , et al. Over expression of long non‐coding rna panda promotes hepatocellular carcinoma by inhibiting senescence associated inflammatory factor il8. Sci Rep. 2017;7:4186. 28646235 10.1038/s41598-017-04045-5 PMC5482898

[4] Shi X , Sun M , Liu H , et al. Long non‐coding rnas: a new frontier in the study of human diseases. Cancer Lett. 2013;339:159‐166. 23791884 10.1016/j.canlet.2013.06.013

[5] Huang S , He X . The role of micrornas in liver cancer progression. Br J Cancer. 2011;104:235‐240. 21102580 10.1038/sj.bjc.6606010 PMC3031886

[6] Quinn JJ , Chang HY . Unique features of long non‐coding rna biogenesis and function. Nat Rev Genet. 2016;17:47‐62. 26666209 10.1038/nrg.2015.10

[7] Schmitt AM , Chang HY . Long noncoding rnas in cancer pathways. Cancer Cell. 2016;29:452‐463. 27070700 10.1016/j.ccell.2016.03.010 PMC4831138

[8] Gupta RA , Shah N , Wang KC , et al. Long non‐coding rna hot air reprograms chromatin state to promote cancer metastasis. Nature. 2010;464:1071‐1076. 20393566 10.1038/nature08975 PMC3049919

[9] Graham LD , Pedersen SK , Brown GS , et al. Colorectal neoplasia differentially expressed (crnde), a novel gene with elevated expression in colorectal adenomas and adenocarcinomas. Genes Cancer. 2011;2:829‐840. 22393467 10.1177/1947601911431081 PMC3278902

[10] Jiang H , Wang Y , Ai M , et al. Long noncoding rna crnde stabilized by hnrnpul2 accelerates cell proliferation and migration in colorectal carcinoma via activating ras/mapk signaling pathways. Cell Death Dis. 2017;8:e2862. 28594403 10.1038/cddis.2017.258 PMC5520914

[11] Croce CM . Causes and consequences of microrna dysregulation in cancer. Nat Rev Genet. 2009;10:704‐714. 19763153 10.1038/nrg2634 PMC3467096

[12] Wang RT , Xu M , Xu CX , et al. Decreased expression of mir216a contributes to non‐small‐cell lung cancer progression. Clin Cancer Res. 2014;20:4705‐4716. 24958806 10.1158/1078-0432.CCR-14-0517

[13] Duan H , Li Y , Yan L , et al. Microrna‐217 suppresses homocysteine‐induced proliferation and migration of vascular smooth muscle cells via n‐methyl‐d‐aspartic acid receptor inhibition. Clin Exp Pharmacol Physiol. 2016;43:967‐975. 27333430 10.1111/1440-1681.12611

[14] Wei R , Deng Z , Su J . Mir‐217 targeting wnt5a in osteosarcoma functions as a potential tumor suppressor. Biomed Pharmacother. 2015;72:158‐164. 26054690 10.1016/j.biopha.2015.04.012

[15] Giannelli G , Koudelkova P , Dituri F , et al. Role of epithelial to mesenchymal transition in hepatocellular carcinoma. J Hepatol. 2016;65:798‐808. 27212245 10.1016/j.jhep.2016.05.007

[16] Chen JS , Li HS , Huang JQ , et al. Microrna‐379‐5p inhibits tumor invasion and metastasis by targeting fak/akt signaling in hepatocellular carcinoma. Cancer Lett. 2016;375:73‐83. 26944318 10.1016/j.canlet.2016.02.043

[17] Yuan JH , Yang F , Wang F , et al. A long noncoding rna activated by tgf‐beta promotes the invasion‐metastasis cascade in hepatocellular carcinoma. Cancer Cell. 2014;25:666‐681. 24768205 10.1016/j.ccr.2014.03.010

[18] Santarpia L , Lippman SM , El‐Naggar AK . Targeting the mapk‐ras‐raf signaling pathway in cancer therapy. Expert Opin Ther Targets. 2012;16:103‐119. 22239440 10.1517/14728222.2011.645805 PMC3457779

[19] Li XW , Tuergan M , Abulizi G . Expression of mapk1 in cervical cancer and effect of mapk1 gene silencing on epithelial‐mesenchymal transition, invasion and metastasis. Asian Pac J Trop Med. 2015;8:937‐943. 26614994 10.1016/j.apjtm.2015.10.004

[20] Zhang N , Lu C , Chen L . Mir‐217 regulates tumor growth and apoptosis by targeting the mapk signaling pathway in colorectal cancer. Oncol Lett. 2016;12:4589‐4597. 28105166 10.3892/ol.2016.5249 PMC5228443

[21] Ma MZ , Chu BF , Zhang Y , et al. Long non‐coding rna ccat1 promotes gallbladder cancer development via negative modulation of mirna‐218‐5p. Cell Death Dis. 2015;6:e1583. 25569100 10.1038/cddis.2014.541 PMC4669740

[22] Salmena L , Poliseno L , Tay Y , et al. A cerna hypothesis: the rosetta stone of a hidden rna language? Cell. 2011;146:353‐358. 21802130 10.1016/j.cell.2011.07.014 PMC3235919

[23] Chou J , Wang B , Zheng T , et al. Malat1 induced migration and invasion of human breast cancer cells by competitively binding mir‐1 with cdc42. Biochem Biophys Res Commun. 2016;472:262‐269. 26926567 10.1016/j.bbrc.2016.02.102

[24] Wang SH , Wu XC , Zhang MD , et al. Long noncoding rna h19 contributes to gallbladder cancer cell proliferation by modulated mir‐194‐5p targeting akt2. Tumour Biol. 2016;37:9721‐9730. 26803515 10.1007/s13277-016-4852-1

[25] Nie W , Ge HJ , Yang XQ , et al. Lncrna‐uca1 exerts oncogenic functions in non‐small cell lung cancer by targeting mir‐193a‐3p. Cancer Lett. 2016;371:99‐106. 26655272 10.1016/j.canlet.2015.11.024

[26] Sun J , Bie B , Zhang S , et al. Long non‐coding rnas: critical players in hepatocellular carcinoma. Int J Mol Sci. 2014;15:20434‐20448. 25387074 10.3390/ijms151120434 PMC4264176

[27] Lan T , Yan X , Li Z , et al. Long non‐coding rna pvt1 serves as a competing endogenous rna for mir‐186‐5p to promote the tumorigenesis and metastasis of hepatocellular carcinoma. Tumour Biol. 2017;39:1010428317705338. 28656879 10.1177/1010428317705338

[28] Lu S , Zhou J , Sun Y , et al. The noncoding rna hoxd‐as1 is a critical regulator of the metastasis and apoptosis phenotype in human hepatocellular carcinoma. Mol Cancer. 2017;16:125. 28724429 10.1186/s12943-017-0676-x PMC5518122

[29] Liu T , Zhang X , Gao S , et al. Exosomal long noncoding rna crnde‐h as a novel serum‐based biomarker for diagnosis and prognosis of colorectal cancer. Oncotarget. 2016;7:85551‐85563. 27888803 10.18632/oncotarget.13465 PMC5356757

[30] Chen Z , Yu C , Zhan L , et al. Lncrna crnde promotes hepatic carcinoma cell proliferation, migration and invasion by suppressing mir‐384. Am J Cancer Res. 2016;6:2299‐2309. 27822419 PMC5088293

[31] Yang X , Xie X , Xiao YF , et al. The emergence of long non‐coding rnas in the tumorigenesis of hepatocellular carcinoma. Cancer Lett. 2015;360:119‐124. 25721084 10.1016/j.canlet.2015.02.035

[32] Liz J , Esteller M . Lncrnas and micrornas with a role in cancer development. Biochim Biophys Acta. 2016;1859:169‐176. 26149773 10.1016/j.bbagrm.2015.06.015

[33] Tay Y , Rinn J , Pandolfi PP . The multilayered complexity of cerna crosstalk and competition. Nature. 2014;505:344‐352. 24429633 10.1038/nature12986 PMC4113481

[34] Yu B , Ye X , Du Q , et al. The long non‐coding rna crnde promotes colorectal carcinoma progression by competitively binding mir‐217 with tcf7 l2 and enhancing the wnt/beta‐catenin signaling pathway. Cell Physiol Biochem. 2017;41:2489‐2502. 28472810 10.1159/000475941

[35] Lin L , Han MM , Wang F , et al. Cxcr7 stimulates mapk signaling to regulate hepatocellular carcinoma progression. Cell Death Dis. 2014;5:e1488. 25341042 10.1038/cddis.2014.392 PMC4649507

[36] Guegan JP , Ezan F , Theret N , et al. Mapk signaling in cisplatin‐induced death: predominant role of erk1 over erk2 in human hepatocellular carcinoma cells. Carcinogenesis. 2013;34:38‐47. 23042098 10.1093/carcin/bgs317

[37] Yiwei T , Hua H , Hui G , et al. Hotair interacting with mapk1 regulates ovarian cancer skov3 cell proliferation, migration, and invasion. Med Sci Monit. 2015;21:1856‐1863. 26117268 10.12659/MSM.893528 PMC4489685

[38] Zhu M , Chen Q , Liu X , et al. Lncrna h19/mir‐675 axis represses prostate cancer metastasis by targeting tgfbi. FEBS J. 2014;281:3766‐3775. 24988946 10.1111/febs.12902

[39] Huan J , Xing L , Lin Q , et al. Long noncoding rna crnde activates wnt/beta‐catenin signaling pathway through acting as a molecular sponge of microrna‐136 in human breast cancer. Am J Transl Res. 2017;9:1977‐1989. 28469804 PMC5411947

[40] Hermeking H , Rago C , Schuhmacher M , et al. Identification of cdk4 as a target of c‐myc. Proc Natl Acad Sci U S A. 2000;97:2229‐2234. 10688915 10.1073/pnas.050586197 PMC15783

[41] Shi Y , Xu X , Zhang Q , et al. Trna synthetase counteracts c‐myc to develop functional vasculature. Elife. 2014;3:e02349. 24940000 10.7554/eLife.02349 PMC4057782

[42] Fantozzi A , Gruber DC , Pisarsky L , et al. Vegf‐mediated angiogenesis links emt‐induced cancer stemness to tumor initiation. Cancer Res. 2014;74:1566‐1575. 24413534 10.1158/0008-5472.CAN-13-1641

[43] Chen XL , Nam JO , Jean C , et al. Vegf‐induced vascular permeability is mediated by fak. Dev Cell. 2012;22:146‐157. 22264731 10.1016/j.devcel.2011.11.002 PMC3266538

